# Circadian Dysfunction in Adipose Tissue: Chronotherapy in Metabolic Diseases

**DOI:** 10.3390/biology12081077

**Published:** 2023-08-02

**Authors:** Erkan Civelek, Dilek Ozturk Civelek, Yasemin Kubra Akyel, Deniz Kaleli Durman, Alper Okyar

**Affiliations:** 1 Department of Pharmacology, Faculty of Pharmacy, Istanbul University, 34116 Istanbul, Turkey; erkan.civelek@istanbul.edu.tr (E.C.); deniz.kaleli@istanbul.edu.tr (D.K.D.); 2 Department of Pharmacology, Faculty of Pharmacy, Bezmialem Vakıf University, 34093 Istanbul, Turkey; dozturk@bezmialem.edu.tr; 3 Department of Medical Pharmacology, School of Medicine, Istanbul Medipol University, 34815 Istanbul, Turkey; yasemin.akyel@medipol.edu.tr

**Keywords:** circadian dysfunction, adipose tissue, metabolic diseases, obesity, diabetes, chronotherapy

## Abstract

**Simple Summary:**

The circadian timing system is our bodies’ built-in clock and controls our physiology on a daily basis. This system helps us adapt to changes in our environment, such as light and dark cycles, temperature changes, and the timing of meals. Disruptions to this system are linked to many health problems, including cancer, sleep disorders, and metabolic disorders such as diabetes and obesity. One key player in this system is adipose tissue, or fat, which stores and releases energy. Changes in how this tissue works can significantly impact our metabolic health. This article explores how the circadian timing system and adipose tissue interact and how disruptions to this interaction can lead to metabolic diseases. Furthermore, the potential of chronotherapy, a new field that uses our understanding of the circadian timing system to improve treatments for metabolic disorders, is discussed. This approach includes the timing of medication and targeting specific genes that regulate our natural clock. By understanding these complex interactions, it could be possible to develop more effective treatments for metabolic disorders such as obesity and diabetes.

**Abstract:**

Essential for survival and reproduction, the circadian timing system (CTS) regulates adaptation to cyclical changes such as the light/dark cycle, temperature change, and food availability. The regulation of energy homeostasis possesses rhythmic properties that correspond to constantly fluctuating needs for energy production and consumption. Adipose tissue is mainly responsible for energy storage and, thus, operates as one of the principal components of energy homeostasis regulation. In accordance with its roles in energy homeostasis, alterations in adipose tissue’s physiological processes are associated with numerous pathologies, such as obesity and type 2 diabetes. These alterations also include changes in circadian rhythm. In the current review, we aim to summarize the current knowledge regarding the circadian rhythmicity of adipogenesis, lipolysis, adipokine secretion, browning, and non-shivering thermogenesis in adipose tissue and to evaluate possible links between those alterations and metabolic diseases. Based on this evaluation, potential therapeutic approaches, as well as clock genes as potential therapeutic targets, are also discussed in the context of chronotherapy.

## 1. Introduction

The circadian timing system (CTS) is a biological mechanism that regulates the daily rhythmicity of the physiological processes in living organisms. It is a crucial regulator of adaptation to environmental changes, such as the light/dark cycle, temperature fluctuations, and nutrient availability. Epidemiological research has established a correlation between circadian disruption and the development of various health conditions, including cancer, sleep and behavioral disorders, and metabolic disorders such as diabetes and obesity. Energy homeostasis, a fundamental aspect of overall health, demonstrates rhythmic properties that correspond to the body’s dynamic energy demands. As a primary component of the energy regulation system, adipose tissue plays a crucial role in the storage and release of energy as required. Thus, alterations in adipose tissue physiology can have significant effects on metabolic health, including the development of obesity and type 2 diabetes. The importance of comprehending the intricate relationship between the CTS and adipose tissue function is underscored by the fact that circadian rhythm disturbances were linked to these metabolic disorders [1,2,3].

This article seeks to summarize the current understanding of the circadian rhythmicity of important adipose tissue processes, such as adipogenesis, lipolysis, adipokine secretion, browning, and non-shivering thermogenesis. By investigating the complex interaction between circadian rhythm and adipose tissue function, we can elucidate the potential mechanisms underlying metabolic diseases. In addition, we investigate the pathophysiology underlying the implications of circadian rhythm dysfunction in obesity and type 2 diabetes.

Chronotherapy, an emerging field that optimizes therapeutic interventions using the knowledge of circadian rhythms, holds tremendous promise for the treatment of metabolic disorders. In this review, we investigate the potential applications of chronotherapy approaches, such as the timing of anti-diabetic and anti-obesity drugs, in enhancing treatment efficacy and patient outcomes. In addition, we discuss the possibility of targeting clock genes, which are essential regulators of the circadian system, as a novel therapeutic strategy for metabolic diseases.

We aim to provide a comprehensive overview of the complex relationship between circadian rhythm, adipose tissue function, and metabolic diseases by examining the most recent research in this field. In addition, we aim to highlight the therapeutic potential of chronotherapy and clock gene modulation as novel approaches in the field of metabolic disorders. Through a greater comprehension of these intricate mechanisms, we can pave the way for more targeted and effective interventions to combat the growing global burden of obesity and type 2 diabetes. Overall, this review underscores the importance of circadian rhythm in adipose tissue function and highlights the potential for chronotherapy as a novel therapeutic strategy in the field of metabolic disorders.

## 2. Circadian Rhythm

The 24-h daily periods, as a result of the rotation of the earth around its own axis, form the basic framework of the environmental conditions in which all organisms live. Adaptation to cyclical changes such as the light/dark cycle, temperature change, and food availability is crucial for survival and reproduction. This adaptation is mainly carried out by the circadian timing system (CTS), which controls many important biological functions in mammals [4,5,6].

### Molecular Mechanisms of Clock Machinery

**Figure 1:**
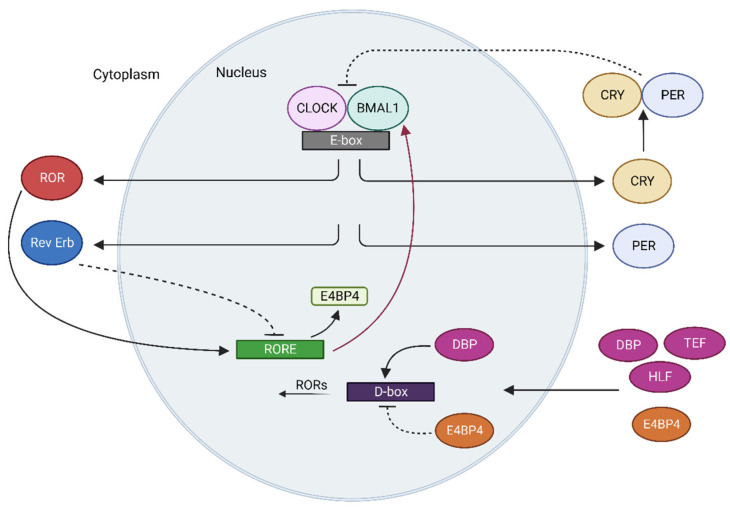
The core clock, secondary, and third interlocked transcriptional feedback loops. CLOCK-BMAL1 are transcription factors and compose a heterodimer that rhythmically activates clock-controlled genes (CCGs) binding E-Box. In turn, PER and CRY translocate into the nucleus and inhibit CLOCK-BMAL1-driven transcription. In the secondary loop, transcription of nuclear receptors RORs and REV-ERBs controls Bmal1 transcription via RORE and regulates the CLOCK-BMAL1 indirectly. DBP-E4BP4 are transcription factors that form a third loop either activates (DBP) or represses (E4BP4) gene transcription from RORs via D-box.

The CTS consists of molecular clocks found in all cells and the suprachiasmatic nucleus (SCN), which undertakes the main clock function that synchronizes these molecular clocks. Cellular molecular clocks can sustain themselves without any regulatory cue (Zeitgeber, ZT) from the external environment and function by interlocking translational–transcriptional feedback loops. Two transcription factors, namely, muscle aryl hydrocarbon receptor nuclear translocator-like protein 1 (BMAL1) and circadian locomotor output cycles kaput (CLOCK; clock circadian regulator for humans); CRY1 and CRY2 cryptochromes; *PER1*, *PER2*, *PER3* period genes play a key role in cellular oscillation [6]. BMAL1 and CLOCK form a heterodimer and bind to the promoter regions of the *PER* and *CRY* genes, activating their transcription. The resulting PER and CRY proteins are also coupled to form a heterodimer, similar to BMAL1 and CLOCK. The PER:CRY structure physically interacts with BMAL1:CLOCK and causes the heterodimer to dissociate from the promoter region [7]. In this context, the negative feedback generated by the PER:CRY heterodimer serves as a measure of cellular circadian time. The new cycle starts with the degradation of the PER and CRY proteins. The PER protein is degraded by the ubiquitin-proteasome pathway [8,9,10,11]. Similarly, adenosine-monophosphate-activated protein kinase (AMPK) and glycogen synthase kinase 3 beta (GSK3β) were shown to be responsible for the degradation of CRY1 and CRY2 proteins, respectively [12,13]. Cells also have auxiliary mechanisms such as reverse-erythroblastosis virus alpha/beta (REV-ERBα/β) and retinoic-acid-receptor-related orphan receptors (RORα/β/γ) in addition to this basic oscillation mechanism. The BMAL1:CLOCK heterodimer structure increases transcription by binding to the promoter regions of the *REV-ERB* [14] and *ROR* genes [15]. The resulting protein products, REV-ERBs and RORs, compete with each other for binding to the promoter region of the *BMAL1* gene. RORs increase *BMAL1* gene transcription while REV-ERBs suppress it [16]. Other auxiliary oscillation mechanisms, in addition to REV-ERBs and RORs, include differentiated embryonic chondrocytes 1 and 2 (DEC1/2), leucine zipper transcription factor E4 promoter-binding protein 4 (E4BP4), and proline- and acid-rich basic leucine zipper transcription factor albumin d-box binding protein (DBP) [17,18]. The core clock, secondary, and third interlocked transcriptional feedback loops are shown in Figure 1.

## 3. The Role of Circadian Rhythm in Adipose Tissue Function

**Figure 2:**
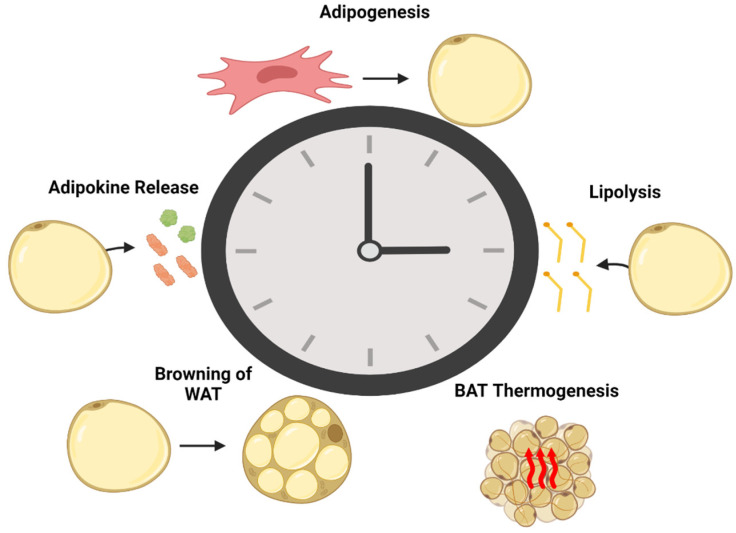
The circadian regulation of adipogenesis, lipolysis, brown adipose tissue (BAT) thermogenesis, browning of white adipose tissue (WAT), and adipokine release. The circadian clock, represented by the central clock machinery, influences the timing and coordination of adipose tissue functions throughout the day. The central role of the circadian clock in orchestrating the rhythmicity of adipose tissue processes highlights the importance of circadian regulation in maintaining metabolic balance and its potential implications for metabolic disorders.

This section discusses the effects of circadian rhythm on adipose tissue processes such as adipogenesis, lipolysis, adipokine secretion, browning, and non-flickering thermogenesis. It is illustrated in Figure 2.

### 3.1. The Role of Circadian Rhythm in Adipogenesis

Adipogenesis, which refers to the de novo formation of adipocytes from multipotent mesenchymal precursors, is one of the major adaptive mechanisms to confer a positive energy balance. Adipogenesis is comprised of two consecutive phases. The first phase, in which bone morphogenetic protein (BMP) signaling plays a critical role, is characterized by the determination of mesenchymal precursor cells to pre-adipocytes. In the latter stage, the committed pre-adipocytes differentiate to mature adipocytes under the control of peroxisome proliferator-activated receptor-γ (PPARγ), the master regulator of adipogenesis, and transcription co-activators CCAAT/enhancer-binding protein α and β (C/EBPα and C/EBPβ) [19]. Upon activation, PPARγ and C/EBPα initiate the adipogenic program that includes adipogenic-specific proteins such as glucose transporter 4 (GLUT4), lipoprotein lipase (LPL), stearoyl CoA desaturase-1 (SCD1), phosphoenol pyruvate carboxykinase (PEPCK), and fatty acid binding protein 4 (FABP4) [20].

Considering food intake is a cue for the circadian rhythm itself at both the cellular and organism levels and adipogenic hormones such as glucocorticoids have oscillatory patterns of secretion, it is not surprising that adipogenesis has rhythmic properties consistent with the fact that PPARγ, the master regulator of adipogenesis, has a circadian expression pattern in white adipose tissue (WAT) [21,22]. However, cautious consideration is needed when linking the rhythmicity of glucocorticoid secretion and adipogenesis. The pulsatile manner of glucocorticoid secretion does not seem to resonate with adipogenesis, as Bahrami-Nejad et al. showed that a transcriptional circuit, possibly via fast and slow positive feedback mechanisms upon PPARγ, filters glucocorticoid oscillations to induce adipogenesis in adipocyte precursor cells [23].

The molecular circadian clock machinery has a substantial role in adipogenesis. In a study, it was shown that BMAL1 has a crucial role in adipogenesis, BMAL1 mRNA is highly expressed in differentiated 3T3-L1 cells, and the *Bmal1*-deficient mice embryonic fibroblast cells failed to differentiate into 3T3-L1 cells [24]. Researchers also reported that PPARγ agonist partially but not fully restores the differentiation potential of *Bmal1* deficient cells, suggesting BMAL1 does not have total control over PPARγ and the relation between two pose coordination. Consistent with their previous study, *Bmal1* KO mice had a diminished capacity for fat storage, as well as decreased expression levels of marker genes for mature adipocytes [25]. Conversely, BMAL1 is a negative regulator of adipogenesis via the Wnt pathway, which is known to repress adipogenesis [26]. Based on these results, although BMAL1 plays a role in adipogenesis, the direction of the effect is not conclusive. Furthermore, none of the aforementioned studies were conducted in human cells (e.g., human adipose-derived stem cells), thus, the role of BMAL1 in human adipogenesis is currently unknown.

In addition to BMAL1, PER3 was also shown to have a role in adipogenesis, both in vivo and in vitro. The primary mesenchymal stem cells (MSCs) isolated from *Per3* KO mice had an increased level of adipogenesis compared to MSCs from WT mice, suggesting the inhibitory effect of PER3 on adipogenesis. PER3 directly interacts with PPARγ and inhibits its activity [27]. The possible inhibitory effect of PER3 is also supported by evidence from a study that reported an increased level of adipogenesis in adipocyte precursor cells from *Per3* KO mice [28]. The proposed mechanism is the regulation of Kruppel-like factor 15 (KLF15), which induces PPARγ expression [29]. The effect of the post-transcriptional modification of PER3 on adipogenesis was also defined. Accordingly, mIR-181a was shown to target *PER3* and induce adipogenesis, further supporting the inhibitory role of PER3 [30].

Auxiliary oscillators, which offer fine-tuning for the circadian rhythm, were shown to have a role in adipogenesis. It was demonstrated that BMAL1 suppressor (*Nr1d1*, the REV-ERBα gene) has a biphasic mRNA and protein expression profile during adipogenesis in mice 3T3-L1 cells. Interestingly, the protein expression profile is opposite to the gene expression profile of REV-ERBα chronologically [31]. Continuous light exposure increased adipogenesis in zebrafish larvae with the mRNA expression of REV-ERBα, further supporting the involvement of REV-ERBα in adipogenesis [32]. In contrast to REV-ERBα, RORα was shown to suppress adipogenesis at a later stage via induction of the expression of DEC1 and DEC2 in 3T3-L1 adipocytes [33]. The inhibitory effect of RORα was suggested to be mediated by the inhibition of the transcriptional activity of CEBP/β, resulting in the inhibition of PPARγ [34]. SREBP-1c was also proposed as a mechanistic link between RORα and adipogenesis [35]. Melanoma antigen family member D1 (MAGED1), which interacts with RORα, was demonstrated to negatively regulate adipogenesis [36]. In addition to REV-ERBs and RORs, DBP has a significant role in adipogenesis, as Suzuki and colleagues demonstrated in the knock-down model for *DBP* gene, which had a decreased level of adipocyte differentiation with downregulated PPARγ expression [37].

In addition to the molecular circadian clock components, genes regulated by the circadian machinery are shown to affect adipogenesis. Nocturnin, which is a deadenylase, is shown to be upregulated in differentiated adipocytes [38]. A further study, which reported that nocturnin stimulates PPARγ translocation, provides a mechanistic link between nocturnin and adipogenesis [39]. Angiopoietin-like 2 (Angptl2), whose expression has circadian rhythmicity similar to nocturnin, has a role in adipogenesis. Kitazawa et al. demonstrated that *Angptl2* siRNA inhibited adipogenesis in 3T3-L1 preadipocytes [40]. Inhibitor of DNA binding 2 (ID2) is another circadian protein involved in adipogenesis. ID2 overexpression increased PPARγ expression in 3T3-L1 preadipocytes [41].

Even though PPARγ does not have circadian rhythmicity in brown adipose tissue (BAT) [22], adipogenesis is affected by the cellular clock in BAT in mice. BMAL1 is shown to inhibit brown adipogenesis in both the commitment and differentiation stages, possibly via transforming growth factor beta (TGF-β) and BMP signaling pathways [42].

### 3.2. The Role of the Circadian Rhythm in Adipose Tissue Lipolysis

The fatty acids released after lipolysis are the primary source of energy for many organs and tissues, especially skeletal muscles. In adipose tissue, lipolysis is mainly controlled by three key hydrolytic enzymes, namely, hormone-sensitive lipase (HSL), monoglyceride lipase (MGL), and adipose triglyceride lipase (ATGL). In addition to this, the post-transcriptional and post-translational regulation of these hydrolases is well characterized. Furthermore, other components of hydrolytic machinery (e.g., regulatory proteins such as comparative gene identification-58 (CGI-58), G0/G1 switch gene-2 (G0S2), and hypoxia-induced lipid-droplet-associated protein (HILPDA)) have important functions in terms of the regulation of lipolysis in adipose tissue [43].

Given that physical activity is concentrated at certain times of the day evolutionarily, the need for a daily rhythm in energy expenditure is reasonable. As expected, lipolysis in both adipocytes [44] and adipose tissue possess circadian rhythm [45,46,47,48]. Both the central clock and peripheral clocks seem to have significant roles in this rhythmicity [47,49,50]. In a randomized clinical trial, altering the circadian rhythm by mild cold exposure increases lipolysis associated with increased plasma levels of fibroblast growth factor 21 (FGF21), indicating the remarkable role of the central component of circadian rhythmicity [49]. Nocturnin is also proposed as a link between the central clock and epididymal adipose tissue lipolysis in mice. Nocturnin expression is not rhythmic in the epididymal WAT of mice housed at 12 h/12 h light–dark cycle and fed ad libitum. cAMP, which is also an inducer of lipolysis, induces nocturnin mRNA expression in restricted-fed mice. Thus, nocturnin may serve as an indicator of the extent of lipolysis linked to the overall energy intake of the body [51]. Hormone secretion, which is an output of the central clock, is another modulator of lipolysis in adipose tissue. In a clinical study, suppression of the early morning cortisol rise by metyrapone increased adipose LPL activity and decreased HSL activity and lipolysis in subcutaneous adipose tissue, showing the link between the central clock and WAT lipolysis via cortisol [52]. Another hormone linking the central clock to lipolysis is growth hormone (GH). Boyle and colleagues demonstrated that during sleep, blood glycerol concentration, which is used as a measure of lipolysis, decreases continuously in GH-deficient subjects. In contrast, glycerol levels initially decreased and then increased in normal subjects [53]. In accordance with the aforementioned studies showing that the central clock affects lipolysis, several studies showed interventions affecting the central clock alter lipolytic pattern. Bartness reported fat mass loss induced by short-day in Siberian hamsters [54]. Similarly, cold exposure was shown to alter lipolysis [49]. In a randomized controlled clinical trial in which all the factors affecting the central clock such as sleep, light exposure, energy intake, and physical activity were controlled, late eating resulted in a shift towards adipogenesis rather than lipolysis [55]. In addition to eating time, the timing of physical activity also affects lipolysis. Kato et al. demonstrated that exercise in a late period of the active phase increases isoproterenol-induced lipolysis and HSL protein expression in male Wistar rats. In addition, researchers observed that late-time exercise increased the association of BMAL1 with protein kinase A (PKA) regulatory units, AKAP150, which is the anchoring protein of PKA, and HSL showing a mechanistic link between the circadian clock and lipolysis [56].

Cellular clocks and factors affecting molecular clocks in adipose tissue contribute to the regulation of lipolysis. Natriuretic peptide A (NPA) and C (NPC) receptor expressions are shown to be associated clock genes in 129/Sy mice adipose tissue and NPA receptor expression was found to correlate with plasma free fatty acid (FFA) levels [50]. In another study, Noshiro and colleagues showed disrupted circadian rhythmicity of lipolytic genes expressions in adipose tissue of *Dec1* deficient mice [57]. Aryl hydrocarbon receptor (AhR) agonist β-napthoflavone (BNF) was shown to decrease lipolysis via suppressing clock gene and lipolysis gene transcription levels in mice adipocytes [58]. Early growth response 1 (EGR1), which is a target for insulin and also has a rhythmic expression pattern in adipocytes, inhibits ATGL and lipolysis [59].

The relationship between circadian rhythm and lipolysis is generally believed to be unidirectional. Nonetheless, studies indicate that lipolysis itself can regulate circadian clock machinery. By using machine-learning algorithms, Markussen and colleagues reported that lipolysis regulates the circadian clock machinery. Upon stimulation, lipolysis overrules the rhythmicity of clock genes and establishes a new rhythm [60]. By the same token, the pharmacological inhibition of lipolysis normalizes impaired nocturnal GH secretion and pulsatility in obese patients [61].

Several studies show that the circadian rhythm of lipolysis is disrupted in obesity. Adipose tissue lipolytic activity in overweight women does not differ between morning and evening [62]. In obese type 2 diabetic patients, the rhythmic expression of genes involved in lipolysis is impaired [63]. In contrast, Amador and colleagues observed/had shown rhythmicity for HSL in the abdominal adipose tissue of morbidly obese patients. However, only HSL activity and HSL transcript expression levels were measured in this study; plasma lipid and glycerol levels were not [64]. In addition to obesity, the circadian rhythmicity of lipid metabolism is impaired in cancer cachexia [65].

In addition to adipocytes, the regulation of lipolysis is influenced by cellular clocks in other cell types. *Bmal1* deficiency in visceral adipose tissue regulatory T cells (Treg) disrupts the rhythm of lipolysis, suggesting the modulatory role of the immune system in the circadian regulation of lipolysis [66]. Furthermore, intestine specific *Bmal1* deficiency is reported to have profound effects on glucose and lipid metabolism [67].

### 3.3. Adipokine Release

Adipokines secreted from adipose tissue have many effects on various physiological processes, especially the metabolism. The circadian regulation of metabolic processes such as food intake and energy expenditure extend to the adipokine secretion profile. Leptin, which, among other physiological effects, acts as a satiety signal, has a rhythmic secretion profile in both animals and humans [68,69,70,71,72,73]. In a clinical trial, it was observed that leptin pulse amplitude and 24-h leptin plasma levels are higher in women than men. Rhythmic patterns, on the other hand, do not differ between sexes [69]. This pattern of rhythmicity is governed by the central component of the CTS, as Kalsbeek and colleagues showed that leptin rhythm is controlled by the central clock and neither cortisol, feeding, nor insulin play a role in the regulation of leptin rhythm in rats [71]. Similarly, SCN is the main determinant and constant glucocorticoid release, or different feeding regimes do not alter leptin rhythmicity in Syrian hamsters [74]. On the contrary, starvation abolishes the leptin rhythm, and there is a causal link between food intake and the rhythm of leptin secretion in humans [75]. Similar findings in terms of the effect of starvation on leptin rhythmicity were also reported in male Wistar rats [76]. Furthermore, the consumption of carbohydrates with a high glycemic index was found to change the rhythmicity of leptin secretion [77]. The role of the central clock was also shown in an animal study in which ventromedial hypothalamus (VMH) lesions in rats were shown to alter the leptin secretion rhythm, showing the significant role of the central clock [78]. Studies investigating the possible hormonal regulation of leptin rhythm demonstrated that similar to cortisol [74], LH [79] and GH [80] are not associated with leptin secretion. However, prolactin is proposed as a possible regulator of nocturnal leptin secretion [81].

The rhythm of leptin secretion is impaired in many conditions. Studies show that acute sepsis [82], depression in young females [83], heroin abstinence [84], aging [85], and increased BMI [86] in humans impair rhythmic leptin secretion. However, the leptin profile did not change in either Cushing syndrome [87] or in stage IV cancer patients [88]. In animal studies, obesity [89], stress [89,90], and aging [91] are associated with altered patterns of leptin secretion. The mechanistic link between the aforementioned conditions and alterations in the rhythm of leptin secretion remains largely unknown.

Adiponectin is another adipokine that was extensively studied in terms of the circadian rhythm. Studies investigating the possible rhythmic profile of adiponectin secretion have conflicting results. Gavrila and colleagues reported that adiponectin has ultradian pulsatility and diurnal rhythm in healthy men. The further evidence supporting the notion of circadian regulation of adiponectin secretion derives from studies showing vasoactive intestinal peptide (VIP), which is shown to regulate feeding behavior, deficient mice have altered adiponectin secretion [92]. In addition, MAGED1, which interacts with RORα, deficiency increases adiponectin levels [36]. Moreover, the rhythm of adiponectin secretion was shown to be impaired in obesity [93] and hyperinsulinemia [94]. In line with the aforementioned studies, *Bmal1* deficient mice have higher levels of adiponectin compared to WT controls [95], implying the circadian regulation of adiponectin secretion. On the contrary, several studies reported that adiponectin has no circadian rhythmicity. A study shows the circadian expression profile of adiponectin mRNA in the rat adipose tissue depot, but plasma levels of adiponectin do not exhibit rhythmic characteristics [90]. Similarly, in a murine cell culture study, adiponectin secretion did not have circadian rhythmicity [96]. Similar results were obtained from both animal [97,98] and clinical [99,100] studies. Other adipokines, such as visfatin [101,102,103], vaspin [104], retinol binding protein 4 (RBP4) [105], apelin [106], and resistin [107] were shown to have a rhythmic secretion profile. Additionally, shift work was found to result in elevated serum resistin [108,109]. On the other hand, omentin [110] and chemerin [111] do not exhibit rhythmicity in terms of plasma levels.

### 3.4. Brown Adipose Tissue Non-Shivering Thermogenesis

Adaptation to the ambient temperature is a vital function for all mammals. Since temperature changes throughout the day and night, thermogenesis is expected to align with temperature changes to maintain homeostasis. BAT is the site responsible for non-shivering thermogenesis via the uncoupling of electrons from the ATP synthesis process in the mitochondria [112]. In concordance with the rhythm of the ambient temperature, BAT thermogenesis is shown to have a daily rhythm [113,114,115]. In addition, studies in which thermogenesis was not directly measured demonstrated that genes and proteins related to thermogenesis in BAT had a rhythmic expression profile [116,117]. The central component of the rhythm regulation was shown in several studies. VMH, one of the nuclei connected to SCN in hypothalamus, was demonstrated to control BAT thermogenesis in mice. Interestingly, the specific deletion of *Bmal1* in VMH neurons is sufficient to disrupt BAT thermogenesis, showing that even though BAT thermogenesis has circadian rhythmicity, the source of the rhythm is the cellular rhythms in VMH rather than SCN [118]. Similarly, Felipe and colleagues showed that food deprivation and cold altered the circadian pattern of BAT glycogen content, which is considered an indicator of BAT thermogenesis. However, it was not determined whether the effect of cold or food deprivation was mediated through the circadian clock system or any other mechanisms [119]. Peripheral clocks are also involved in the regulation of the rhythmicity of BAT thermogenesis. Angers and colleagues demonstrated that major facilitator superfamily domain-containing protein 2a (Mfsd2a) is upregulated in BAT during thermogenesis via β adrenergic signaling [120]. Much clearer evidence regarding the role of the cellular clocks in BAT thermogenesis is presented in the study of Lee and colleagues. In this study, GLUT4, UCP1, and REV-ERBα expression rhythms related to thermogenesis were reported in human brown adipocytes [114]. However, central control of the rhythm of BAT thermogenesis seems to be dominant over the cellular clocks, as shown in a study that demonstrates that BAT-specific deletion of *Bmal1* only has a mild effect on thermogenesis [121]. The possible rhythm of the adaptive thermogenic response to cold was also investigated, but conflicting results were reported. In a randomized controlled clinical trial, cold-induced thermogenesis was reported not to have diurnal variation [122]. On the other hand, as indirect evidence, Machado and colleagues showed that cold-induced alterations in thermogenesis-related gene expressions have diurnal variation [123]. Furthermore, Straat and colleagues demonstrated diurnal variation in cold-induced thermogenesis in only males, suggesting sexual dimorphism [124].

Aside from rhythmicity, thermogenesis in BAT is influenced by stimuli that affect the circadian clock system. For instance, a short photo period stimulates thermogenesis in rodents [125,126,127]. The further research revealed that thermogenesis is entrained in changed light periods [128]. Adaptive thermogenic responses to diet and cold exposure were also studied extensively. In genetically obese ob/ob mice, food restriction causes increased BAT thermogenesis [129]. However, Eley and colleagues reported the suppression of thermogenesis by food restriction in lean but not obese rats [113]. Cold-induced thermogenesis is decreased in *Per2* deficiency [130] and increased in *Nr1d1* genetic loss [131,132]. Interestingly, *Bmal1* deficiency did not alter the thermogenic capacity according to the study of Li and colleagues [133]. The deficiency of *Dec1*, an oscillatory component of the circadian clock, enhances thermogenic *Ucp1* gene expression [134]. In addition, the deletion of circadian-regulated ID2 was shown to cause increased glucose uptake by the BAT [135]. The hormonal mediators of circadian time are also of importance. Corticotropin-releasing factor was shown to induce BAT thermogenesis. Similarly, melatonin deficiency decreases the level of BAT thermogenesis. Interestingly, Viswanathan and colleagues reported that pinealectomy did not influence BAT thermogenesis [136]. It can be suggested that there is no overall hormonal influence from the hypothalamus–pituitary axis. According to the aforementioned conflicting results, it is not possible to determine whether temperature, feeding, or photic stimuli are the primary exogenous cues of time. Further well-designed, comprehensive studies, especially utilizing BAT-specific deletion of clock genes, are needed.

### 3.5. Browning of Adipose Tissue

Adipocytes are known to have plasticity according to the metabolic needs of the organism [137,138]. Stimuli such as thyroxine, β-3 adrenergic signaling, cold exposure, etc., inform adipose tissue of the need to readjust the balance between energy storage and utilization [139]. Thus, white adipocytes may undergo a phenotypic change toward brown adipocytes, a process known as “browning”. The resulting “beige or brite” adipocytes have increased UCP1 protein levels, which are responsible for thermogenesis via the uncoupling of electrons from the ATP synthesis in the inner membrane of the mitochondria. Beige adipocytes may also arise from progenitor cells in adipose tissue [140].

Similar to adipogenesis, the browning process is shown to have rhythmic properties and be related to circadian clock machinery. Grimaldi and colleagues demonstrated that PER2 is a suppressor of the browning process and that *Per2* deficiency activates the browning process in the WAT of mice via PPAR-dependent genes. The researchers further observed that PPARγ interacts with the *Ucp1* PPAR response element on the genome in *Per2* deficient mice in contrast to wild-type controls [141]. Similarly, the browning process was reported to be induced in the WAT of Rora-deficient mice. Browning in these mice was induced through PR domain zinc finger protein 16 (PRDM16) and PPAR-gamma coactivator 1-alpha (PGC-1α). Nonetheless, PPARγ expression was not measured in this study [142]. Even though BMAL1 is known to suppress the brown adipogenesis, the involvement of BMAL1 in the browning process was not investigated by using *Bmal1* KO models. FGF21 [143], an inducer of browning, has a diurnal rhythm that is modulated by cold exposure, providing additional evidence regarding the rhythm of adipose tissue browning [49]. Altering the circadian rhythm in rats by phase shifting caused reduced UCP1 mRNA expression levels in brown adipose tissue and metabolic risk. However, linking these results to the studies showing shift workers have altered circadian rhythmicity and impaired metabolic profile requires cautious consideration. The mechanisms by which altered rhythmicity impairs the metabolic profile in humans are poorly understood and need further investigation. Particular attention should be paid to the potential effects of altered immune system activity on metabolic parameters in shift workers.

## 4. Circadian Rhythm Dysfunction in Obesity and Type 2 Diabetes

The disruption of the circadian clock is shown to be related to a variety of diseases, including psychiatric disorders, sleep disturbances, and metabolic disorders such as obesity, type 2 diabetes, metabolic syndrome, and cardiovascular disease [11,144,145]. Shift work and social jetlag are considered to be common factors causing circadian disruption and resulting in sleep disturbances, as well as metabolic disorders [146]. Sleeping for less than 6 h or more than 9 h is linked to a higher risk of developing type 2 diabetes and impaired glucose tolerance [147,148]. According to the research, shift workers have an increased risk of developing type 2 diabetes, and the number of work nights seems to be an important factor regarding the circadian disruption [145,149,150]. Furthermore, patients with type 2 diabetes who worked night shifts had worse glycemic control compared to day workers [151].

In a human study, it was seen that insufficient sleep causes human adipocytes to become insulin-resistant, indicating sleep as a significant factor for energy homeostasis in adipose tissue. After four nights of sleep deprivation compared to regular sleep, cellular insulin sensitivity in adipocytes from subcutaneous fat samples reduced by ~30% in healthy, young, lean men and women [152].

Sleep deprivation may also lead to reduced leptin levels and an increase in the hormone ghrelin, which both regulate appetite. Whereas leptin, which is mostly released by adipose tissue, suppresses appetite, ghrelin increases food intake, decreases fat oxidation, and consequently, causes an increase in adiposity. Leptin and ghrelin may be insufficient to appropriately express calorie requirements as a result of sleep deprivation, and their combined effects may lead to the false impression of a limited energy supply [153,154]. In a clinical study when healthy subjects were exposed to 88 h of sleep deprivation with scheduled meals, the leptin diurnal rhythm amplitude showed a significant decrease. In the following recovery period, leptin amplitude significantly increased, indicating sleep loss’s effect on the daily leptin rhythm [155]. Furthermore, the nocturnal ghrelin increase is shown to be blunted by sleep loss [156].

As discussed above, genetic KO mice of core circadian clock genes have altered lipid metabolism. Furthermore, the extensive knowledge showed a strong link between the circadian disruption and metabolic diseases. Adipose-*Bmal1* knockout and liver- and adipose- *Bmal1* KO mice show altered feeding behavior and locomotor activity compared with WT mice. When liver- and adipose-*Bmal1* KO mice were fed a diabetogenic diet, their body weight and adiposity increased compared to WT animals [157]. Brown adipocyte-specific *Bmal1* KO (BA-*Bmal1* KO) mice did not show a change in their body temperature due to an increase in locomotor activity and shivering, but the diurnal rhythmicity of fatty acid use in BAT was disrupted, and both BAT thermogenesis and total body energy expenditure were somewhat decreased. BA-*Bmal1* KO mice were also found to be more susceptible to obesity when given a diet high in fat [121]. *Clock* mutation is also known to affect feeding behavior and energy homeostasis. The current research shows that mice with the *Clock* mutation have increased weight gain, hyperphagia, increased adiposity, and adipocyte hypertrophy, as well as glucose and lipid metabolism disorders [158].

In addition to the effect of the circadian clock disruption on the metabolic disorders, the dysregulation of energy homeostasis in metabolic disorders may affect the circadian rhythms, directly or indirectly. In a study in male mice, high-fat-diet (HFD)-induced obesity caused significant changes in the expression of core circadian clock genes; clock-controlled targets related to lipid metabolism in the hypothalamus, the liver and the adipose tissue; and also, significant variations observed in the locomotor activity rhythm. The results of this study indicated that the diurnal rhythm was dampened, and the circadian period was lengthened in mice on a high-fat diet [159]. Conversely, in another study in female mice fed a high-fat diet and having mild metabolic syndrome, the thigh-fat diet had minor effects on the circadian rhythm of core clock genes in the adipose tissue and the liver, while the cholesterol 7a-hydroxylase rhythm was dampened, indicating that peripheral circadian rhythms may be altered [160]. In another study by Ando et al., the rhythmic expression of *Bmal1*, *Per1*, *Per2*, *Cry1*, *Cry2*, *Dbp*, and adipocytokines such as adiponectin, resistin, and visfatin in visceral adipose tissue weakened in obese and obese-diabetic mice with a superior effect on obese-diabetic animals. When obese-diabetic mice were treated with pioglitazone, the rhythmicity of the clock genes showed improvement in the liver tissue [161]. Additionally, obesity causes disruption of the adipocyte clock via inhibition of PPARγ, which regulates adipogenesis and leads to the downregulation of *Bmal1* and other clock genes. As a result, the progression of obesity and circadian disturbance interact bidirectionally [162].

Moreover, there are some clinical studies evaluating the relationship between metabolic disorders and circadian rhythm dysfunction in adipose tissue. In a study in morbidly obese patients, the expression levels of the circadian clock genes in adipose tissue were shown to be related to the metabolic syndrome markers [163]. In another study comparing lean, overweight/obese, and overweight/obese-type 2 diabetic patients under high levels of glycemic control, the rhythmic gene expression of core clock genes and metabolic genes in white adipose tissue did not display significant changes depending on the metabolic state [164]. Santos et al. evaluated the circadian expression of clock genes in subcutaneous and visceral adipose tissue cultures of female morbidly obese patients and found that the circadian rhythm was maintained ex vivo for a minimum of two circadian cycles following surgery, indicating the presence of the cellular circadian clock that controls individual cellular processes separate from the SCN. In addition, according to correlation analyses of the circadian rhythm and metabolic syndrome markers, having a larger sagittal abdominal diameter leads to an increase in circadian variability of Per2 and Bmal1 gene expression [165]. *PER2* and *NR1D1* expressions in subcutaneous adipose tissue of overweight subjects were shown to be increased after body weight loss with a caloric restriction [166].

## 5. Chronotherapy Approaches in Metabolic Disorders

Chronotherapy is an approach to treatment that considers the circadian rhythm of the body and employs timing interventions to maximize efficacy and minimize adverse effects. Chronotherapy can be used to treat sleep disorders, mood disorders, and certain types of cancer. In the management of diabetes and obesity, chronotherapy may entail synchronizing the timing of meals, physical activity, and drug administration with the body’s natural circadian rhythm to improve blood sugar regulation. In addition, it may entail modifying meal timing and composition to optimize metabolism and energy balance. Interruptions in the circadian rhythm, such as those caused by shift work or irregular sleep patterns, can alter insulin secretion and increase the risk of metabolic disorders.

### 5.1. Chronotherapy of Antidiabetic and Anti-Obesity Drugs

Chronotherapy is an emerging field of study that aims to optimize the timing of medication administration so that it corresponds more closely with the body’s natural insulin sensitivity and glucose metabolic patterns. Metformin, a commonly prescribed anti-diabetic medication, was investigated in the context of chronotherapy, and research suggests that evening administration may result in superior glycemic control compared to morning administration. These studies have demonstrated that metformin has a complex effect on the circadian clock, with effects on the liver and muscle, and that the acute decrease in blood glucose in response to metformin therapy is time dependent. Metformin reduces body weight gain, normalizes glucose tolerance and insulin resistance markers, and restores islet morphology and cell apoptosis more effectively when combined with melatonin in a rat model of HFD and circadian-disruption-induced obesity (CDO) [167]. In addition, metformin increases leptin levels while decreasing glucagon levels and leads to the activation of AMPK by liver kinase B1 (LKB1) and/or other muscle kinases, resulting in phase advances in the liver and phase delays in the muscle in the expression of clock genes and/or metabolic proteins [168]. Metformin activates casein kinase I epsilon (CKI epsilon) by activation of AMPK, which, in turn, leads to mPer2 degradation and a phase advance in the circadian expression pattern of clock genes in the peripheral tissues of WT mice, but not in AMPK alpha2-deficient mice, and decreases the circadian period of Rat-1 fibroblasts by one hour [169]. Henriksson et al. demonstrated that the time of day influences the instantaneous reduction of blood glucose in response to metformin treatment and blood lactate levels in healthy mice and that ablation of Bmal1 expression in the liver modifies, but does not completely eliminate, diurnal variations in AMPK and blood glucose responses induced by metformin [170]. Alex et al. examined the effect of metformin on diabetic retinopathy (DR). Metformin therapy restored the expressions of retinal clock genes and Kir4.1 in the retina, which were downregulated in DR. Additionally, they showed that metformin upregulated clock regulating genes and AMPK activation via metformin-boosted Kir4.1 and Bmal1 expression in rMC-1 cells. Silencing AMPK1 lowered the protein expression of Kir4.1 and BMAL1 [171]. Metformin restores AMPK-SIRT1 signaling and WAT circadian function in db/db and HFD mice by increasing AMPK activity and, consequently, the expression of NAMPT, SIRT1, and circadian components. Metformin drives a phenotypic shift away from fat accumulation via AMPK-NAMPT-SIRT1-mediated alterations in clock components, providing support for chronotherapeutic approaches to the treatment of obesity [172]. Additionally, metformin’s human pharmacokinetics are affected by interindividual variation and time of day due to oscillations in glomerular filtration rate (GFR), renal plasma flow (RPF), and organic cation transporter 2 (OCT2) activity, indicating that the individual chronotype may be important for metformin’s chronotherapy [173].

PPARs were found to be involved in the regulation of circadian rhythm and metabolism, and recent studies have revealed that all PPARs carry out their functions in a circadian manner [174]. Bezafibrate, a ligand of PPARα, accelerated the active phase of mice under light/dark (LD) conditions in a photoperiod-dependent way, suggesting that PPARα is involved in the synchronization of the circadian clock to LD environmental circumstances. Additionally, PPARα is involved in photo entrainment of the circadian clock without affecting the circadian period [175,176]. A midnight injection of bezafibrate significantly stimulated PPARα-dependent FGF21 expression, which is crucial for adaptations to fasting, including lipolysis and ketogenesis, but a daytime infusion had little impact. Furthermore, PPARα-deficient mice lacked the circadian FGF21 expression induced by bezafibrate [177]. Prolonged injection of the PPARα ligand Wy14643 significantly reduces body temperature and suppresses nighttime locomotor activity [178]. The body temperature of mice treated with bezafibrate declined late at night, and this may have contributed to the lower late night behavioral activity under LD 8:16 [179].

Rosiglitazone and pioglitazone, which are PPARγ agonists, are shown to increase insulin sensitivity and are used to treat type 2 diabetes. PPARγ exhibits a circadian expression pattern in the liver, fat, and blood vessels of mice, implying that it plays a crucial role in the regulation of the circadian clock. These results demonstrate that PPAR is an indispensable regulator of adipogenesis and a well-established therapeutic target for the treatment of metabolic diseases. Yang et al. observed that pioglitazone, PPARγ improved physiological parameters and reversed the majority of circadian-clock gene expression changes in the liver in a non-obese insulin resistance mice model [180]. Rosiglitazone improved the disrupted liver clock in the same experimental model. They also showed that PPARγ antagonist GW6662 reversed the positive effects of rosiglitasone on the liver clock [181]. Ribas-Latre et al. investigated the effects of a HFD on the recruitment of the circadian transcription factor BMAL1 in metabolically active tissues and found that restoring whole body insulin sensitivity with rosiglitazone was sufficient to restore changes in BMAL1 recruitment and activity [182]. Interestingly, in one study, the rhythmicity of clock genes and adipokines in perigonadal adipose tissues was attenuated in obese KK and more obese, diabetic KK-A(y) mice and further impaired by a 2-weeks treatment of pioglitazone, although it improved the attenuated rhythmicity in the liver [161]. Reversed feeding disrupted the mouse liver circadian expression pattern of clock genes and increased inflammatory markers, but administration of pioglitazone restored the clock gene expression profile and decreased inflammation. Pioglitazone intake at 7 PM was more effective than at 7 AM in reversed feeding mice, thus, pioglitazone had a potent chronopharmacological effect when administered at 7 PM to mice [183].

There are also a few studies on other antidiabetic drugs. Hennessey et al. found that administering glyburide before bedtime was more effective than in the morning for individuals with Type 2 diabetes, resulting in improved fasting blood sugar and carbohydrate tolerance curves without any hypoglycemia [184]. The diurnal effects of sitagliptin induced anti-hyperglycemia in obese mice. Sitagliptin administration in the light phase significantly decreased plasma glucose levels, insulin levels, hepatic steatosis, and restored glucose tolerance in comparison to the HFD group, indicating that sitagliptin displays definite chronopharmacology [185].

There are studies and ongoing research examining the combination of chronotherapy and anti-obesity medications. The goal is to optimize the timing of medication administration so that it is more in sync with the circadian rhythm and metabolic processes of the body to enhance weight loss and metabolic outcomes. Bromocriptine affects circadian rhythms, and it was hypothesized to reset hypothalamic circadian activities that were altered by obesity, thereby reversing insulin resistance and reducing glucose production. Bromocriptine-QR, a formulation with rapid release, was shown in clinical trials to improve glycemic control and reduce postprandial hyperglycemia [186,187,188] and reduce adverse cardiovascular events in type 2 diabetes patients [189]. Preclinical studies suggest that the mechanisms of action include amelioration of the loss of responsiveness of hypothalamic glucose-sensing neurons to hyperglycemia [190], reduction of elevated sympathetic tone [191], reduction of leptin resistance [191,192,193], and reduction of MBH NPY and AgRP mRNA expressions [194]. Moreover, bromocriptine influences several MBH genes associated with neuronal plasticity [194].

In two Italian studies, the efficacy of fenfluramine chronotherapy in the treatment of obesity was evaluated. The results indicated that administering a single dose of 80 mg in the morning for four weeks led to a greater decrease in weight and adipose mass than administering the drug in the afternoon or three times a day [195]. In addition, this administration was associated with a shorter duration of consumption, a lower total calorie intake, and a reduction in appetite [196]. These findings suggest that morning administration of fenfluramine chronotherapy may be an effective treatment for obesity. Drugs that affect hypothalamic pathways may play a role in the chronotherapy of metabolic diseases as well. Oxytocin release in the hypothalamus displays a diurnal rhythm that is disrupted by chronic high-fat-diet feeding, and the manipulation of oxytocin can be used to reprogram energy expenditure and control obesity. A peripheral injection of oxytocin can also activate hypothalamic oxytocin neurons to exert metabolic effects, providing a potential clinical avenue for obesity control in mice [197]. Lee and Bray found that patients with hypothalamic obesity had altered mechanisms controlling insulin secretion when compared to obese patients without hypothalamic injury, including a lack of diurnal variation in glucose-stimulated insulin secretion and an inability to be affected by naloxone. Naloxone increased insulin sensitivity in the obese control patients but had no effect on patients with hypothalamic obesity or normal weight subjects [198]. In a study that examined the effects of nicotine on glucose metabolism in db/db mice with type 2 diabetes, it was found that oral nicotine consumption increased hypothalamic prepro-orexin gene expression and decreased hyperglycemia without affecting body weight, body fat content, or insulin serum levels. It also revealed that nicotine reduced the mRNA levels of glucose-6-phosphatase, the rate-limiting enzyme of gluconeogenesis, in the livers of db/db and WT mice [199].

### 5.2. Targeting the Circadian Clock in Metabolic Diseases

The disruption of circadian rhythms is linked to metabolic disorders. Targeting these clock genes may be the key to treating metabolic diseases.

Obesity is associated with disruption of the circadian clock in WAT in both rodents and humans. Whang et al. found that a significant decrease in BMAL1 expression in WAT may be the link. In the study, the decreased expression of PPARγ in obese WAT trans-activates the uptake transporter Slc1a5; impaired PPARγ in obesity leads to the downregulation of SLC1A5 and decreased adipocyte uptake of glutamine and methionine (two epigenetic modulators), which disrupts Bmal1 [162]. Nakata et al. explored the role of Bmal1 in the hypothalamic paraventricular nucleus (PVN) in glucose metabolism. The deletion of *Bmal1* in the paraventricular nucleus (PVN) of the hypothalamus decreased insulin secretion, leading to impaired glucose tolerance. It was also discovered that fasting conditions inhibit arginine vasopressin (AVP) expression in *Bmal1* KO mice, suggesting that PVN BMAL1 maintains AVP expression and its release into the circulation, helping enhance insulin release and glucose tolerance. However, the circadian variation of AVP expression is regulated by feeding but not by PVN BMAL1 [200]. Mandl et al. revealed the role of BMAL2 in human adipose stem/progenitor cells (ASCs). BMAL2 functions as an inhibitor of both the mechanistic target of rapamycin (mTOR) and mitogen-activated protein kinase (MAPK) signaling pathways, leading to a feedback mechanism. A Western blot analysis of sWAT samples from normal-weight, obese, and weight-loss (WL) donors revealed that the BMAL2 protein was solely elevated by WL compared to BMAL1; demonstrating that BMAL2 is a WL-regulated adipogenesis inhibitor may aid in the development of strategies to combat obesity [201].

REV-ERBα/β are important nuclear receptors that regulate energy balance and adiposity, and their disruption can lead to increased food intake and decreased energy expenditure, resulting in diet-induced obesity. In male mice lacking circadian nuclear receptors REV-ERBα/β in the tuberal hypothalamus, REV-ERB-dependent leptin signaling in the arcuate nucleus was impaired, leading to impaired diurnal leptin sensitivity. These KO mice fed an obesogenic high-fat diet acquired excessive weight due to decreased energy expenditure and increased food intake during the light phase [202]. Synthetic REV-ERB agonists were identified that can alter the circadian pattern of core clock gene expression in rodents, and when administered to mice with diet-induced obesity, obesity was reduced, and metabolic diseases were alleviated [203]. Furthermore, REV-ERBα was identified as a new intracellular regulator of glucagon secretion in alpha-cells. High glucose levels inhibit key genes controlled by AMPK, such as Nampt, Sirt1, and PGC-1α, and Rev-erbα expression. AMPK activation by metformin can reverse the inhibitory effect of glucose on Nampt-Sirt1-PGC-1α and Rev-erbα, leading to increased glucagon secretion [204]. Garaulet et al. found an association between the REV-ERB-ALPHA1 rs2314339 genotype and obesity in two independent populations. Minor allele carriers had a lower probability of abdominal obesity than noncarriers. Additionally, physical activity, but not energy intake, significantly differed by genotype. This discovery highlights the importance of REV-ERB-ALPHA1 in obesity and provides evidence for the connection between our biological clock and obesity-related traits [205].

RORα-deficient mice exhibit a lean and obesity-resistant phenotype due to increased Ucp1 expression in BAT and subcutaneous WAT. This is linked to the increased expression of thermogenic genes, and these mice maintain greater thermal control and cold tolerance relative to their WT littermates [206]. Another study investigating the function of RORα in thermogenesis and the browning of white and brown adipose tissue in RORα-deficient mice demonstrated that RORα acts as an inhibitor of the thermogenic program in white adipose tissue and that RORα antagonists can counteract this role in vivo. Inhibitors of browning differentiation, such as TLE3 and RIP140, could be new RORα targets implicated in the whitened appearance of adipocytes [142]. RORα and RORγ control the activation of SREBP1c to regulate the lipogenic response to feeding. The loss of RORα/γ exacerbates diet-induced hepatic steatosis by activating the SREBP-dependent lipogenic response to feeding to an excessive degree. This highlights the importance of considering the time of day when treating liver metabolic disorders [207].

Lozano et al. investigated the relationship between evening chronotype, obesity, and weight loss in severely obese bariatric surgery patients. There was a significant interaction between the CLOCK 3111T/C SNP and body weight among carriers of the risk allele C, with evening types having a higher body weight than morning types [208]. According to Espinosa-Salinas et al., the rs3749474 CLOCK polymorphism may influence the effects of appetite on waist circumference, with risk allele carriers increasing their waist circumference by 14 cm for each increase in appetite level [209]. Oishi et al. investigated the role of CLOCK in the obesity-induced elevation of plasminogen activator inhibitor-1 (PAI-1). They discovered that CLOCK is involved in obesity-induced disordered fibrinolysis by tissue-dependently regulating PAI-1 gene expression [210].

The deletion of *Cry1/2* results in behavioral and molecular circadian arrhythmicity and increased vulnerability to high-fat-diet-induced obesity, which is mediated by increased insulin secretion and lipid storage in adipose tissues in *Cry1/2*^(-/-)^ mice [211]. In another study, the ablation of *Cry1*, but not *Cry2*, prevented HFD-induced obesity in mice, suggesting increased energy expenditure [212]. These studies reinforce the important role of circadian clock genes in energy homeostasis and suggest that *Cry1* is a plausible target for anti-obesity therapy. Concerning macroautophagy, it affects the circadian clock by selectively degrading CRY1, which occurs in a diurnal window when rodents rely on gluconeogenesis. Mutational analyses have identified two distinct light chain 3 (LC3)-interacting region (LIR) motifs on CRY1 that regulate CRY1 degradation, providing potential targets for controlling hyperglycemia [213].

As potential treatments for metabolic disorders, small-molecule modulators of the circadian clock were investigated in many studies. These modulators interact with the circadian clock genes and can help treat metabolic disorders by modulating the clock. According to studies, small-molecule modulators can reduce the risk of obesity, diabetes, and other metabolic disorders. Additionally, they can enhance sleep quality and reduce fatigue. Small-molecule modulators are a promising treatment option for metabolic disorders and may provide a safe and effective way to improve metabolic health. Small molecules targeting circadian clock proteins have shown therapeutic potential in metabolic diseases. KL001, KL101, and TH301 lengthen the period of the circadian clock and increase brown adipocyte differentiation. TW68, GSK4112, and SR8278 alter circadian gene expression and have metabolic effects such as lowering blood glucose levels and inhibiting gluconeogenesis. SR9009 and SR9011 also alter circadian gene expression and promote increased energy expenditure and decreased fat mass. SR1078 activates ROR-dependent transcription and lowers aerobic glycolysis. Nobiletin enhances the amplitude and lengthens the period of the circadian clock, restoring energy homeostasis and improving metabolic fitness. These small molecules offer potential therapeutic strategies for metabolic diseases by targeting the circadian clock and modulating metabolic processes. Recent evaluations of these molecules were conducted by Kavakli et al. [214], Chen et al. [215], and Rodrigues et al. [216]. Here, we provide a summary of the molecules associated with only metabolic diseases in Table 1.

## 6. Non-Pharmacological Approaches: Time-Restricted Feeding and Light Therapy 

The recent research has demonstrated the tremendous effects of circadian rhythms on metabolic state, the benefits of intermittent fasting, and their relation to the timing of energy intake [231,232]. Through synergistic interactions between the circadian oscillator and feeding–fasting signals, anabolic and catabolic processes are coordinated in accordance with the animal’s activity and rest cycle [233]. Time-restricted feeding, which means limiting eating to the active phase may decrease the predisposition to metabolic diseases and was demonstrated as a nonpharmacological strategy against obesity and diabetes [234,235,236]. However, eating at the “wrong time of day” can have a reverse impact on weight gain and overall metabolic health [237]. Time-restricted feeding, i.e., 8–9 h of food access during the active phase, in C57BL/6 male mice decreased adipose tissue inflammation and changed adipokine levels while reducing and reversing the adiposity caused by obesogenic diets [235]. In a study, mice were subjected to either unrestricted or temporally restricted feeding of a high-fat diet over a duration of 8 h daily, the mice that underwent time-restricted feeding exhibited an augmentation in thermogenesis. Additionally, there was an observed enhancement in the circadian rhythms of core clock genes such as *PER2*, *BMAL1*, *REV-ERBA*, *CRY1*, as well as their target genes. This improvement in circadian rhythms was associated with a protective effect against obesity, hyperinsulinemia, and inflammation [236]. The results of animal studies on time-restricted eating showing increased metabolic health, including lipid parameters and blood glucose, as well as significant weight loss, are also supported by clinical studies in human subjects [238]. In a study in humans, overweight adults under early time-restricted feeding between 8 a.m. to 2 p.m. showed improved 24-h glucose levels, lipid metabolism, and circadian clock gene expression compared to control subjects who ate between 8 a.m. and 8 p.m. [239].

Light therapy, which involves exposing patients to bright light in the early morning for several days, is thought to work by entraining the sleep–wake cycle by ocular stimulation of the suprachiasmatic nucleus and is expected to alleviate sleep disturbances and improve circadian rhythmicity [240,241]. However, in some clinical studies, light therapy was not found to be superior regarding insulin sensitivity, glucose tolerance, and inflammatory parameters, although the peripheral blood mononuclear cells of patients under light therapy showed a significant alteration in the circadian clock gene expressions [241,242].

## 7. Conclusions

In conclusion, CTS plays a crucial role in regulating the functions of adipose tissue and maintaining energy homeostasis. This review has highlighted the significant impact of the circadian rhythm on various processes within adipose tissue, including adipogenesis, lipolysis, adipokine secretion, browning, and non-shivering thermogenesis. Disruptions in these circadian rhythms are associated with metabolic diseases such as obesity and type 2 diabetes. Understanding the intricate relationship between circadian rhythm and adipose tissue function provides valuable insights into the pathogenesis of these disorders. Furthermore, the concept of chronotherapy, which utilizes the knowledge of circadian rhythms to optimize therapeutic interventions, holds promise for the treatment of metabolic diseases. By targeting the circadian clock through chronotherapy approaches, including the timing of antidiabetic and anti-obesity medications, it may be possible to enhance treatment efficacy and improve patient outcomes. Additionally, clock genes emerge as potential therapeutic targets, offering new avenues for intervention in metabolic disorders. Further research and clinical studies are warranted to explore the full potential of chronotherapy and clock gene modulation in the management of metabolic diseases.

## Figures and Tables

**Table 1:** Small molecules targeting circadian clock proteins may have therapeutic potential in metabolic diseases.

Compound	Effects on the Circadian Clock	Mechanism of Action	Metabolic Effects	Refs.
KL001	Period lengthening Amplitude dampening	CRY stabilizer	Represses the induction of gluconeogenesis by glucagon	[217,218]
KL101	Period lengthening	CRY1-selective stabilizer	Increases brown adipocyte differentiation	[219]
TH301	Period lengthening	CRY2-selective stabilizer	Increases brown adipocyte differentiation	[219]
TW68	Period lengthening	CRY Stabilizer	Blood glucose lowering effect in ob/ob mice	[220]
GSK4112	Altering circadian gene expression Represses transcription of Bmal1	REV-ERBα agonist	Inhibits gluconeogenesis	[221]
SR8278	Altering circadian gene expression Increases BMAL1	REV-ERBα antagonist	Inhibits glucagon secretion	[204,222]
SR9009 and SR9011	Altering circadian gene expression Represses transcription of Bmal1	REV-ERBα agonists	Increases energy expenditure and decreases fat mass, plasma triglycerides, and cholesterol levels in diet-induced obesity mouse model. SR9009 inhibits de novo lipogenesis.	[203,223]
SR1078	Activating ROR dependenttranscription	RORα agonist	Lowers aerobic glycolysis. Lowers expression of pyruvate dehydrogenase kinase 2. Inhibits phosphorylation of pyruvate dehydrogenase and promotes the full oxidation of pyruvate.	[224,225]
SR3335	NA	RORα inverse agonist	Inhibits gluconeogenesis and reduces glucose plasma levels.	[226]
SR1555	NA	RORc inverse agonist	Improves insulin sensitivity and decreases food intake in obese diabetic mice. Induces thermogenic gene expression in fat depots, inhibits hormone-sensitive lipase activation, and increases fatty acid oxidation.	[227]
Nobiletin	Amplitude enhancer Period lengthening	ROR agonist	Restores energy hemostasis and prevents metabolic syndrome in mice with diet-induced obesity and db/db mutations. Restores energy hemostasis, improves metabolic fitness, and increases energy expenditure, cold tolerance, exercise endurance, grip strength, and inflammatory markers in aged mice fed a high-fat diet.	[228,229]
Nobiletin	Amplitude enhancer	ROR agonist	Enhances basal and stimulated insulin secretion by T2D islets.	[230]

T2D: type 2 diabetes, NA: not applicable.

## Data Availability

Not applicable.
